# HBV, HCV, and HDV Triple-Infection—A Therapeutic Challenge

**DOI:** 10.3390/diseases13060168

**Published:** 2025-05-26

**Authors:** Alexia Anastasia Stefania Balta, Mariana Daniela Ignat, Raisa Eloise Barbu, Liliana Baroiu, Lavinia Alexandra Moroianu, Valerii Lutenco, Valentin Bulza, Mihaela Patriciu, Caterina Dumitru, Mihaela Debita

**Affiliations:** 1 Doctoral School of Biomedical Sciences, ‘Dunarea de Jos’ University, 800008 Galati, Romania; alexiaanastasia1998@yahoo.com (A.A.S.B.); valerii.lutenco@ugal.ro (V.L.); valibulza@gmail.com (V.B.); patriciu_mihaela@yahoo.com (M.P.); 2 Medical Department, Faculty of Medicineand Pharmacy, ‘Dunarea de Jos’ University, 800008 Galati, Romania; debita_mihaela@yahoo.com; 3 ‘Sf. Apostol Andrei’ Clinical Emergency County Hospital, 800578 Galati, Romania; 4 Clinical Medical Department, Faculty of Medicineand Pharmacy, ‘Dunarea de Jos’ University, 800008 Galati, Romania; lilibaroiu@yahoo.com; 5 ‘Sf. Cuv. Parascheva’ Clinical Hospital of Infectious Diseases, 800179 Galati, Romania; dumitrukati@gmail.com; 6 ‘Sf. Ioan’ Clinical Hospital for Children, 800487 Galati, Romania; 7 Department of Pharmaceutical Sciences, Faculty of Medicine and Pharmacy, ‘Dunarea de Jos’ University, 800008 Galati, Romania; lavinia.moroianu@yahoo.com; 8 Clinical Hospital of Psychiatry “Elisabeta Doamna”, 800179 Galați, Romania; 9 Clinical Surgical Department, Faculty of Medicine and Pharmacy, ‘Dunarea de Jos’ University, 800008 Galati, Romania; 10 Galati Railways General Hospital, 800225 Galati, Romania

**Keywords:** triple hepatitis infection, HBV, HCV, HDV, treatment considerations

## Abstract

Purpose: This article aims to harmonize the current data from the literature, describe baseline severity, and discuss potential treatment considerations for cases of triple infection. Patients and Methods: We undertook a retrospective, observational study on 1244 patients with viral hepatitis study subgroups: chronic replicative hepatitis with HCV—679 patients, HBV—98 patients, HBV/HCV—25 patients, HBV/HDV—14 patients, and 2 patients with triple-infection (HBV, HCV, and HDV), hospitalized in the Second Department of “Sf. Cuv. Parascheva” Infectious Diseases Clinical Hospital of Galați, Romania, between 1 April 2017 and 1 March 2025. Results: Comparative analysis of biochemical parameters and liver fibrosis—at the initial testing—i.e., at the beginning of the specific antiviral therapy—with direct-acting antivirals on HCV (DAAs) or nucleos(t)ide analogues (NUCs): Entecavir (ETV) or Tenofovir Disoproxyl fumarate (TDF), for HBV, Bulevirtide (BLV) for HDV—revealed clinical forms with higher severity in the case of triple and double infections, in comparison to individuals who have had only one hepatotropic virus infection. Conclusions: Compared to patients with a single hepatotropic viral infection, those with a double or triple infection had more severe hepatic damage. Concomitant therapy with Bulevirtide, DAAs, and NUCs is possible and the therapeutic results from clinical studies, with single-infection patients showing great potential for improving the prognosis of these patients.

## 1. Introduction

Acute and chronic viral hepatitis, regardless of their etiology, cause approximately 1.34 million deaths annually throughout the world, remaining a major public health concern [1].

Chronic concomitant infection with the three major hepatotropic viruses—hepatitis B (HBV) virus, hepatitis C (HCV) virus, and hepatitis D (HDV) virus—is rare in low-endemicity areas for HBV and HCV single-infections and more commonly found in high-endemicity areas for these single-infections [2,3,4]. Simultaneous or successive infection with these three hepatitis viruses is possible due to their common transmission route through blood, blood derivatives, contaminated biological fluids, or sexual contact with an infected individual. The results from clinical studies emphasize a more severe and faster clinical evolution towards liver cirrhosis [5] and liver cancer in triple infections, compared to single infections [6]. Therefore, earlier diagnosis and treatment of this triple infection remain a challenge for clinicians, especially due to the fact that European, American, and Asia–Pacific guidelines do not provide clear treatment recommendations.

This article’s goal is to provide a summary of findings from our own clinical experience and research on patients with triple infection (HBV, HCV, and HDV) in order to suggest a diagnostic and therapy protocol for these situations.

### 1.1. Epidemiology of HBV, HCV, and HDV Triple Infection

Although there are case reports from low-endemicity nations as well, many triple-infection cases have been documented in regions with high endemicity for chronic hepatitis with HBV, HDV, and HCV.

For instance, HBV and HDV infection is hyperendemic in Mongolia, Pakistan, Central Asia, Sub-Saharan Africa, the Pacific Islands, the Amazon Basin, and Eastern Europe, especially Moldova. This infection is less common in North America, North Europe, and Japan [2,7,8]. Regarding HCV, the highest numbers of viremic individuals reported in 2015 were in China, Pakistan, India, Uzbekistan, Japan, Kazakhstan, Taiwan, and South Korea [9].

Thirty percent of 207 patients, in 2005, according to a study in Mongolia, were found positive for viremia for all three viruses (HCV, HDV, and HBV) [4]. One patient (0.87%) with a triple viral infection was found in the 1998 China Study, which included 114 patients who were scheduled for surgery [10]. Sixty cases of triple infection with HBV, HCV, and HDV were reported in a 1998 study conducted in Taiwan [11]. In 2000, India reported two cases of triple infection (HBV-DNA positive, HCV-RNA positive, HDV antibodies (HDVAb) positive) in two liver transplant recipients [12]. In 2008, a study from Pakistan analyzed 29 patients with HBV surface antigen (HBsAg), HCV antibodies (HCVAb), and HDVAb positive, of which four cases presented HBV-DNA, HCV-RNA, and HDVAb positive [13]. In 2012, Germany reported a therapeutic success with Pegylated interferon α (IFNα) and Ribavirin in a patient with HBV-DNA, HCV-RNA, and HDVAb positive [14]. In 2017, Poland reported two cases of triple infection and emphasized the resistance to Pegylated interferon, Ribavirin, and Lamivudine of the triple infection with hepatotropic viruses [15].

A meta-analysis of studies on triple infection has observed a high prevalence rate and a three-fold increase in the likelihood of HDV infection among patients with co-infection (HBV/HCV) [6].

A 2024 English study, conducted with the purpose of determining the percentage of patients with chronic hepatitis B eligible for antiviral treatment according to current recommendations, found that, in a group of 7558 adults with chronic hepatitis with HBV, 111 patients are eligible for treatment with NUC due to coinfections with HIV/HCV/HVD [16].

The Polaris 2022 study, conducted in 170 countries with the aim of estimating the global prevalence of HBV infection, approximated 257.5 million individuals with positive HBsAg, of whom only 36 million were diagnosed and 6.8 million had received NUC treatment [17].

### 1.2. Pathogenesis of Triple Infection with HBV, HCV, and HDV

#### Clinical Interaction

Even though they are uncommon, triple infections—HBV, HCV, and HDV—are linked to high severity and have a higher chance of progressing to hepatocellular carcinoma and liver cirrhosis than single infections [6].

In cases of triple infection, one of the viruses is usually dominant. In dual HBV–HDV infections, HDV is dominant in 69.28% of cases; HBV–HDV codominance occurs in 27.56% of cases, and HBV dominance is observed in only 3.16% of cases, according to a meta-analysis from 2020 [2]. HDV is usually the predominant virus in triple infections, with 10–40% of individuals having detectable HCV-RNA [18,19,20]. According to a different study that analysed the HDV dominance in triple infection, 80% of these individuals had no HBV replication indicators or HCV-RNA in their serum [11].

HDV may prevent HBV replication because HDV Antigen (HDVAg) inhibits host DNA-dependent RNA polymerase II, which is important in HBV replication [21,22].

HCV inhibits HBV reproduction, according to another study, and HDV can occasionally cause HBV antigen seroclearance [6].

Significant variations in the viremia levels of HBV, HCV, and HDV, when all present in a patient at the same time, have also been reported in clinical investigations [5,23].

According to a study by Liaw and colleagues on triple infection with HBV, HCV, and HDV, the newly acquired virus inhibits preexisting viruses [11].

Most of the clinical research highlights that triple hepatitis has a more severe clinical course than single infections [6,24].

Triple infection may raise the risk of fulminant hepatitis with severe acute hepatocyte necrosis, according to some research [25].

Compared to individuals with a single infection, people with triple infection have a worse response to interferon therapy [18,26].

#### Experimental Evidence

An experimental study conducted on animals demonstrated the role of HCV in assembling and secreting HDV infectious particles, which may explain the higher prevalence of HDV among HCV-infected patients [27].

Due to the small number of clinical studies on triple-infection HBV/HCV/HDV and the new possibilities of therapy, we propose in this article to bring data from a center in Southeast Europe, from an area with high endemicity for chronic viral hepatitis, to describe comparatively the initial severity of monoinfections with HBV and HCV, as well as of dual-infections HBV/HDV and HCV/HBV and two clinical cases of triple-infection HBV/HCV/HDV and to discuss potential treatment considerations for cases of triple infection.

## 2. Materials and Methods

### 2.1. Study Design

**Figure 1:**
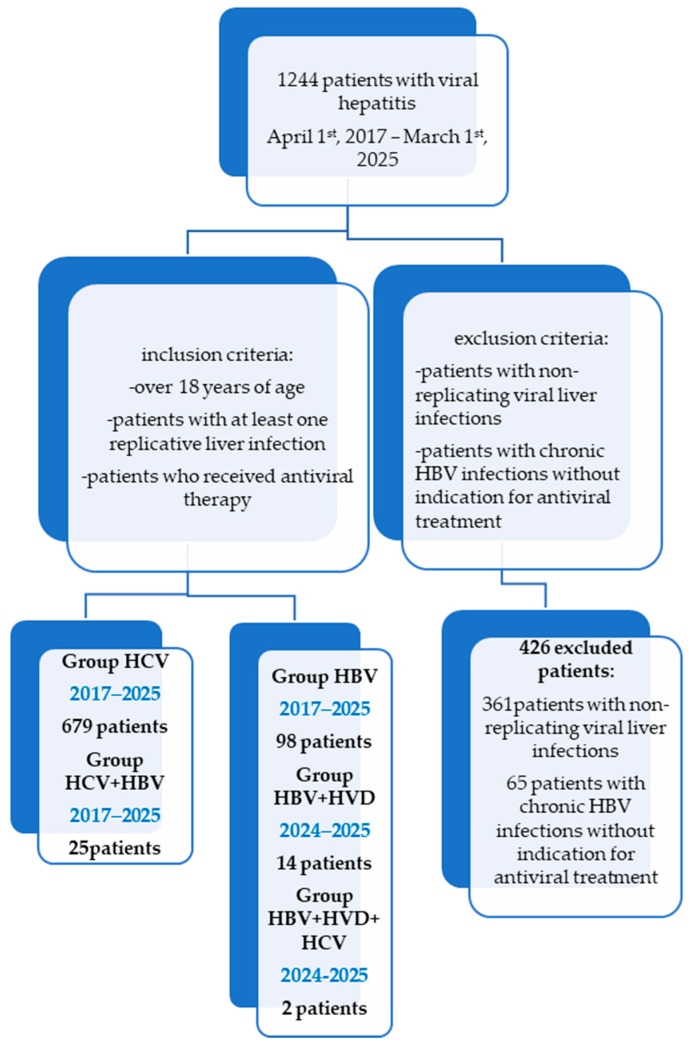
Retrospective chart review-STROBE diagram showing the timeline and different cohorts of patients for a total of 1244 patients treated over an 8-year period of time (2017–2025).

One thousand two hundred forty-four patients with chronic viral hepatitis treated at the Second Department of the “Sf. Cuv. Parascheva” Infectious Diseases Clinical Hospital in Galati, Romania, have been the subject of a retrospective study (Figure 1). The study was conducted following the guidelines of the Declaration of Helsinki and was approved by the Ethics Committee of Dunărea de Jos University of Galați (approval number 43668, on 13 December 2024).

The inclusion criteria were: adults with chronic hepatitis infections with at least one replicative virus who received antiviral treatment between 1 April, 2017, and 1 March, 2025, according to the Romanian criteria for the administration of these therapies at the time of antiviral initiation.

Patients with persistent HBV infections, non-replicative viral liver infections, those without clear indication of needing antiviral therapy, and outliers (those with abnormal laboratory test results for bilirubin, aspartate aminotransferase (AST), alanine aminotransferase (ALT), or international normalized ratio (INR)) were excluded from the study.

Patients with decompensated liver cirrhosis (CHILD [28] B and C or MELD [29] >15) have been excluded from Groups HCV, HBV, and HBV/HVD, as they have received antiviral treatment in a gastroenterology department.

### 2.2. Group Definitions

Subgroups of the study (Figure 1): group HCV comprises 679 patients with a single infection of HCV who were treated with DAAs during the study period. They were provided one of the following combinations: Ledipasvir + Sofosbuvir, Grazoprevir + Elbasvir, Dasabuvir + Ombitasvir + Paritaprevir + Ritonavir, Glecaprevir + Pibrentasvir, Sofosbuvir + Velpatasvir, or Sofosbuvir + Velpatasvir + Voxilaprevir. These patients were HCV-RNA-positive and presented with all kinds of degrees of fibrosis. Patients with active cancer who were undergoing chemotherapy or decompensated liver cirrhosis (CHILD B, C, MELD > 15) have been excluded. It is worth mentioning that, in Romania, Genotype 1b of HCV has been predominant, with a multicenter study from 2017 having noted its presence in 99.6% of patients with chronic hepatitis C and advanced fibrosis from Romania [30,31].

Group HBV contains 98 patients with replicative HBV single infection who started antiviral treatment with Entecavir or Tenofovir Disoproxyl fumarate during the study period, with HBV-DNA > 2000 IU/mL, and at least F1 fibrosis, A1 inflammation (FibroMax), >7 kPa on Fibro Scan, or HBV-DNA positive and F4 on FibroMax/Fibro Scan or with immunosuppressive therapy for other conditions and HBsAg positive (Table 1).

Group HCV/HBV contains 25 patients with co-infection (HCV and HBV) treated with DAA therapy. One of the combinations stated for group HCV has been associated with Entecavir during DAA therapy and for 12 weeks after finishing DAA therapy, if the patients had positive HBsAg but did not meet the antiviral criteria for HBV from group HBV, or they underwent HBV antiviral therapy if they met the criteria for group HBV. Patients with decompensated liver cirrhosis (CHILD B, C, MELD > 15) or active cancer under chemotherapy have been excluded [30,31].

Group HBV/HDV contains 14 patients with replicative co-infection (HBV and HDV) who started Bulevirtide therapy between 1 July, 2024, and 1 March, 2025, which is associated with Entecavir if HBV-DNA > 2000 IU/mL in patients with F0–F3 fibrosis cases or with HBV-DNA positive at any value in patients with F4 fibrosis, CHILD A, and MELD < 15 cases.

Group HBV/HCV/HDV contains two patients with triple infection (HBV, HCV, and HDV) who have received antiviral therapy, which shall be described in detail for each patient in the case studies.

### 2.3. Statistical Plan

It is important to mention that the demographic, biochemical, and fibrosis data analyzed have been collected from the initial evaluation of the patient, which was conducted at the time of antiviral therapy initiation. The study’s objective was to evaluate the severity of dual and triple infections compared to single infections at the time antiviral therapy was started, as well as the percentage of dual and triple infections in an infectious diseases department in Southeast Romania.

Patient data extracted from the observation charts were analyzed using IBM Statistics V. 24 * SPSS, INC., Chicago, IL, USA, and Excel 2019. Descriptive statistics have been calculated for all variables for which this analysis was useful. For continuous numerical variables, minimum, maximum, mean and median value, and standard deviation (SD) have been calculated. For the categorical values, frequency distribution has been calculated. Comparative analysis was performed between the HCV, HBV, HBV/HCV, and HBV/HDV groups with t-test. A statistical significance level of *p* < 0.05 has been considered (with p-index calculated at both ends). The two clinical cases of triple infection will be presented in detail.

The endpoint analysis demonstrated the severity of dual and triple infections (HBV, HCV, HDV), supporting the use of concomitant antiviral therapy in order to improve patient prognosis.

## 3. Results

**Figure 2:**
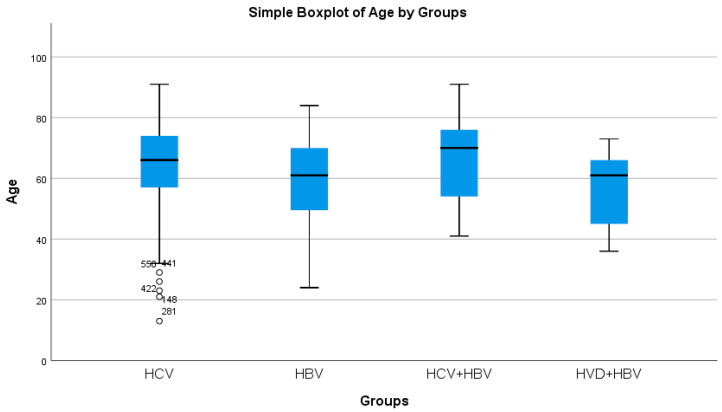
Comparative analysis of the average age (years) in the study groups of patients with hepatic single infections and hepatic co-infections.

Comparative analysis of the parameters at baseline, at the antiviral therapy initiation of the four groups of patients with single infection and co-infection, has revealed the following:

HBV/HCV co-infected patients had the greatest average age, whereas HBV/HDV co-infected patients had the lowest (statistically significant difference, *p* = 0.0214) (Table 2a, Figure 2). Both the HBV/HCV co-infection and HCV single-infection categories were dominated by women (Table 3). Urban background was more common across all study groups (Table 3).

**Figure 3:**
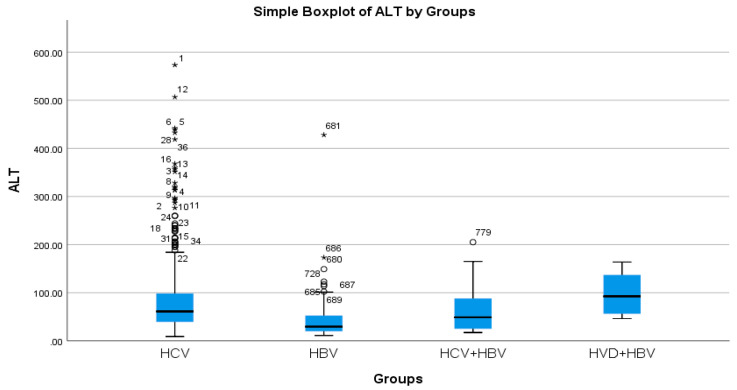
Comparative analysis of the mean ALT (mg/dL) levels in the study groups of patients with hepatic single infections and hepatic co-infections.

**Figure 4:**
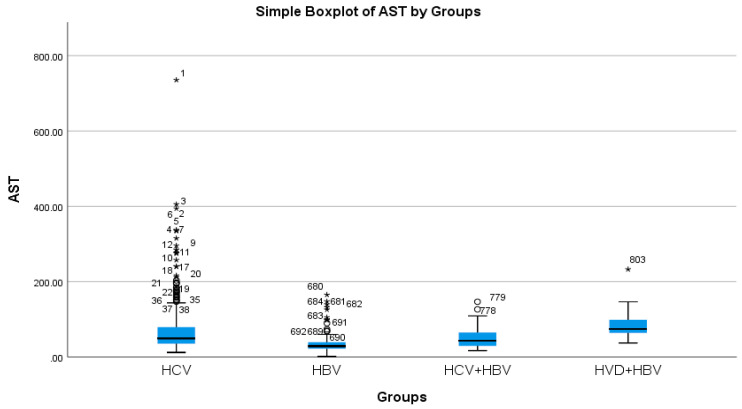
Comparative analysis of the mean AST (mg/dL) levels in the study groups of patients with hepatic single infections and hepatic co-infections.

According to the analysis of the biochemical profile of the liver damage, the group with HBV/HDV co-infection had the highest ALT values (96.27 IU/L), and the group with HBV single infection had the lowest (45.00 IU/L) (Table 2a, Figure 3). There was also a highly significant difference between the two groups (*p* < 0.0001), with the HBV/HDV co-infection group having the highest AST mean value (89.32 IU/L) and the HBV single-infection group having the lowest (41.74 IU/L) (Table 2a, Figure 4).

**Figure 5:**
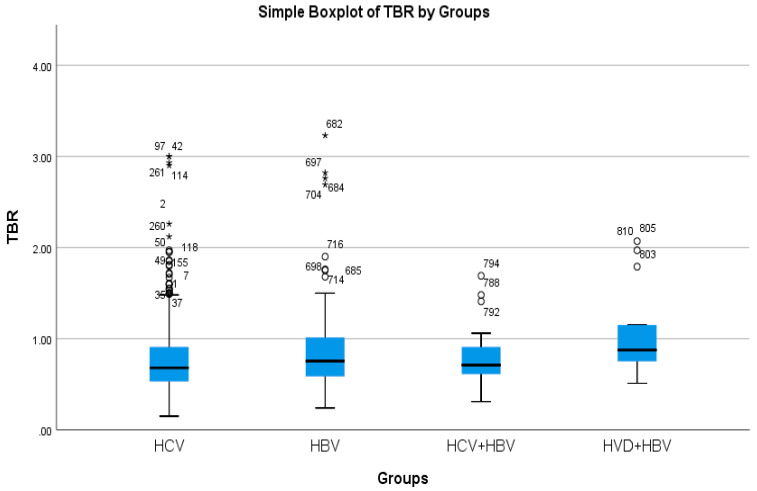
Comparative analysis of the mean TBR (mg/dl×10^2^) levels in the study groups of patients with hepatic single infections and hepatic co-infections.

**Figure 6:**
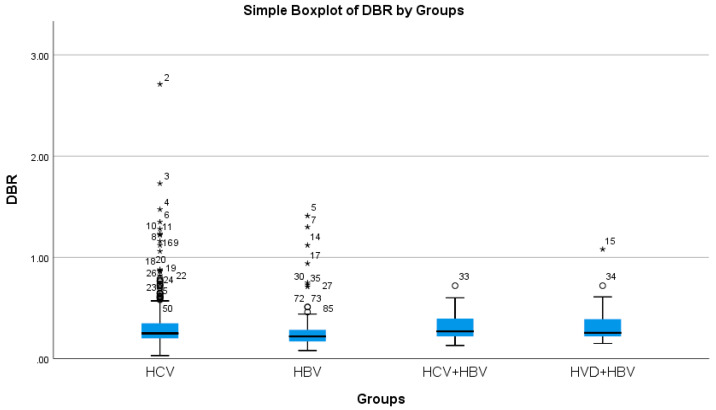
Comparative analysis of the mean DBR (mg/dL mg/dl×10^2^) levels in the study groups of patients with hepatic single infections and hepatic co-infections.

The cholestasis analysis has revealed that the highest total bilirubin was found in HBV/HDV co-infection (1.05 mg/dL), while the lowest was found in HCV single-infection (0.76 mg/dL), with a highly significant difference between them (*p*-value = 0.0032) (Table 2a, Figure 5 and Figure 6).

The analysis of liver’s function of protein synthesis—based on the mean serum albumin values—exhibited the lowest albumin levels in HBV/HDV co-infection (4.27 g/dL), and the highest in the HCV single infection (4.86 g/dL), with a highly significant difference between them (*p*-value = 0.0474) (Table 2b).

The coagulation analysis, based on the mean INR values, exhibited the highest value in the HBV-HDV co-infection group, specifically 1.13, and the lowest in the HCV single-infection group, 1.03, with a highly statistically significant difference between them (*p*-value = 0.0022) (Table 2b). Regarding the mean platelet count in peripheral blood, the lowest mean value of 126.21 × 10^3^/μL had been observed in the HBV-HDV co-infection group, while the highest was 205.29 × 10^3^/μL in the HCV single-infection group, with a highly statistically significant difference between them (*p*-value = 0.0001) (Table 2).

Regarding the mean value of alpha-fetoprotein (AFP)—a key biomarker for liver cirrhosis and hepatocellular carcinoma—the highest mean value among study groups had been observed at 7.36 IU/mL in the cohort with HDV-HBV co-infection, and a minimum value of 3.49 IU/mL between the cohorts, of the HCV-HBV co-infection group, with a statistically significant difference between them (*p*-value = 0.0368) (Table 2).

Hepatic fibrosis assessment at therapy initiation had revealed the predominance of mild and moderate fibrosis (F0, F1, F2) in single-infected groups (HCV: 56.54%, HBV: 67.34%), and the predominance of severe fibrosis (F3, F4) in co-infected groups (HBV/HCV: 72%, HBV/HDV: 78.58%) with a statistically significant difference (*p*-value = 0.0026) between the cohort with the highest severe fibrosis burden (HBV/HDV) and the one with the lowest HBV single infection, with a statistically significant difference (*p*-value = 0.0026) (Table 3).

In the case of HBV, HCV, and HDV triple infection, we present two detailed cases treated in our clinic.

The first is a 57-year-old male who is from an urban area, a carpenter, married with only one sexual partner, tested negative for all three viruses, and with a medical history of dental surgery and blood donation without comorbidities. After receiving a 2001 diagnosis of chronic hepatitis C, the patient had received a year of therapy with pegylated interferon-alpha 2b (PGI) and ribavirin (RIB), and at the end of the course of treatment, they had remained persistently HCV-RNA positive.

He presented to our clinic in September 2024 with HCV, HBV, and HDV compensated liver cirrhosis without other comorbidities. Initial assessment revealed Child–Pugh Class A (5 points), MELD score: 7, FibroMax: F4 (0.89), A3 (0.80), S3 (0.85), N2 (0.75), H0 (0.02), Fibroscan F4 (18.4 kPa) (done on the same day with FibroMax), ALT: 96.4 U/L (normal: 0–45), AST: 52.8 U/L (normal: 0–35), direct bilirubin: 0.29 mg/dL (normal values: 0–0.20), total bilirubin: 0.98 mg/dL (normal values: 0.1–1.2), albuminemia: 4.3 g/dL (normal values: 3.5–5.2), urea: 40.1 mg/dL (normal values: 18–55), creatinine: 0.89 mg/dL (normal values: 0.80–1.30), amylasemia: 72.1 U/L (normal values: 0–100.0), blood glucose: 118.3 mg/dL (normal values: 74–106), total serum cholesterol: 200.1 mg/dL (normal values: 0–200), triglycerides: 285.3 mg/dL (normal values: 0–0.20), INR: 1.03 (normal values: 0.80–1.20), HCVAb positive, HCV-RNA: 1241,115 IU/mL (detection limit: 12 IU/mL), HBsAg: 0.00 (negative 0–0.12), total HBV Total Core Antibodies (HBcAb): 0.11 (negative 1.40–10), HBsAb: 32.2 mIU/ml (negative 0–10.0), HBV-DNA: undetectable (detection limit: 10 IU/mL), HDVAb: positive, HVD-RNA: undetectable (detection limit: 15.15 IU/mL), alpha-fetoprotein: 1.98 IU/mL (normal values: 1–10), complete blood count (CBC): normal, and HIV antibodies (HIVAb): negative. Upper gastrointestinal endoscopy had not revealed esophageal varices, having described mild gastric antral mucosal hyperemia. Abdominal-pelvic ultrasound had shown an anteroposterior diameter of the right hepatic lobe of 145 mm, the left hepatic lobe of 89 mm, portal vein diameter in the hepatic hilus of 11 mm, spleen long axis of 103 mm, and no ascitic fluid. Therapy had been initiated with Glecaprevir (GLE) and Pibrentasvir (PIB) and three tablets of PO QD for 12 weeks, with no side effects. The patient showed ALT and AST normalization within 2 weeks of treatment initiation. Evaluation at 12 weeks post-treatment completion revealed undetectable HCV-RNA, normal ALT, AST, direct and indirect bilirubin and AFP, FIBROMAX-F3: (0.68), A0-A1 (0.21), S2 (0.62), N2 (0.75), and H0 (0.06).

The second is a 75-year-old female who is a rural resident and farmer and was under our department’s observation since December 2017 with HBV, HCV, and HDV-related cirrhosis, with a history of dental surgery, a single spouse, a husband who had passed away at the time of the initial assessment, and comorbidities including degenerative polyarthrosis, chronic pancreatitis, and dyslipidemia.

Initial evaluation revealed: cirrhosis status: CHILD-Pugh Class A (5 points), MELD score: 6 points, Fibroscan: F4 (18.9 KPa), ALT: 81.4 U/L (normal values: 0–45), AST: 65.3 U/L (normal values: 0–35), direct bilirubin: 0.17 mg/dL (normal values: 0–0.20), total bilirubin: 0.55 mg/dL (normal values: 0.1–1.2), albuminemia: 4.45 g/dL (normal values: 3.5–5.2), urea: 57.2 mg/dL (normal values: 18–55), creatinine: 0.98 mg/dL (normal values: 0.80–1.30), amylasemia: 162.5 U/L (normal values: 0–100.0), blood glucose: 100 mg/dL (normal values: 74–106), GGT: 789 U/L (normal values: 0–38), total cholesterol: 261 mg/dL (normal values: 0–200), triglycerides: 164.6 mg/dL (normal values: 0–0.20), INR: 1.02 (normal values: 0.80–1.20), HCVAb: positive, HCV-RNA: undetectable (detection limit: 12 IU/mL), HBsAg: 25.93 (negative: 0–0.12), HBsAb: 66.95 mIU/mL (detection limit: 2.5 mIU/mL), total HBcAb: positive, hepatitis B e antibodies (HBeAb): 0.01 (negative: 0.5–15), hepatitis B e antigen (HBeAg): 0.00 (negative: 0–0.099), HBV-DNA: 8550 IU/mL (detection limit: 10 IU/mL), HDVAb: positive, HDV-RNA: 132,455 IU/mL (detection limit: 15.15 IU/mL), alpha-fetoprotein: 32.14 IU/mL (normal values: 1–10), and complete blood count: normal, HIVAb: negative. Abdominal-pelvic ultrasound had revealed the anteroposterior diameter of the right hepatic lobe of 145 mm, the left hepatic lobe of 50 mm, the caudate lobe of 28 mm, the portal vein diameter in the hepatic hilus of 12.5 mm, the spleen long axis of 110 mm, and no evidence of free intraperitoneal fluid. Therapy had been initiated with Bulevirtide (1 ampoule/day) and Entecavir (0.5 mg/day), with no side effects. Follow-up at 4 weeks showed improvement in hepatic cytolysis (ALT: 53.2 U/L, AST: 53.5 U/L).

## 4. Discussion

In contrast to single infections, double and triple infections with HBV, HCV, and HDV are uncommon but severe, with a greater average biochemical and imaging parameters at the initiation of antiviral treatment, suggesting more severe forms of the disease. There are 0.16% of patients in our department who have triple infections, 1.12% of patients who have been started on antiviral therapy for HBV/HDV co-infection, and 2% of patients who have had HCV/HBV antiviral therapy in our department.

From the perspective of severe liver fibrosis (as measured by Fibroscan scores), hepatic cytolysis (as measured by mean ALT and AST values), cholestasis (as measured by mean BRT values), protein synthesis, and the effect of coagulation (as measured by mean blood albumin, INR, and platelet count), as well as the progression to cirrhosis and HCC (as measured by the mean value of the AFP), our study identifies the most severe forms of the disease at the patients with HBV/HDV co-infection. These patients with HBV/HDV co-infection have the lowest average age and are predominantly male in the urban area.

Advanced stages of fibrosis have also been identified by analyzing the disease severity in patients with co-infection of HBV and HDV.

The use of both parametric (mean ± SD) and non-parametric (median, IQR) descriptors allows a more nuanced interpretation of the data distribution. For example, ALT and AST values in the HBV + HDV group show not only higher means and medians compared to other groups but also increased variability (SD and IQR), reflecting more severe hepatic cytolysis.

High Skewness and Kurtosis in some variables (e.g., AST, TBR) further justify the use of robust statistics and non-parametric tests in comparative analysis, which we have applied and documented accordingly. This descriptive table validates the distribution assumptions used in the main analysis and supports the interpretation of liver injury severity across groups, emphasizing more important liver damage in the group with HBV/HDV co-infection.

INR values show moderate variability across groups, with some evidence of right Skewness, especially in HBV + HDV patients, supporting the presence of coagulopathy. Albumin values remain within normal limits but exhibit slight deviations in the HBV/HDV group with more advanced liver damage. Platelet counts show high variability, particularly in HBV + HDV patients, consistent with portal hypertension and splenic sequestration. High Skewness and Kurtosis values in platelet distribution further emphasize the need for robust statistical methods and confirm the appropriateness of reporting non-parametric measures (median, IQR) for comparative analysis.

A recent study published in Romania [34] has noted that from a group of HBsAg-positive patients (6813), 4.87% presented positive HDV and 75.6% presented a positive HDV-RNA.

The aggressiveness of HDV, the poor response of these instances to interferon, and the relatively recent launch of Bulevirtide therapy in July 2024 are factors that contribute to the discussion of the high severity of this disease. However, we consider that greater fibrosis in HDV co-infection may reflect delayed BLV access rather than intrinsic viral synergy.

Regarding the analysis of parameters in the two patients with triple infection, we note advanced fibrosis (F4) in both cases—one with HCV dominance and the other with HDV dominance.

These triple-infection patients now have new treatment choices thanks to current antiviral therapeutic options, such as bulevirtide for HDV, NUCs for HBV, and DAAs for HCV. The EASL, AASLD, and ASIA-PACIFIC guidelines provide clear evidence and recommendations for treating HBV/HCV and HBV/HDV co-infections, but they do not specify clear guidelines for the treatment of triple infection with these viruses. The expert consensus is to initially treat the dominant virus, the one with the highest replication rate. There is little data in the literature regarding the treatment of these cases, making them a real challenge for every clinician.

Our study highlights statistically significant differences in the increasing severity of the case, in HBV/HDV co-infection, possibly due to the viral aggressiveness of HDV and especially due to the long period during which no specific therapy was available for these patients. It is well known that patients with dual and triple hepatitis virus infections have a poor response to interferon therapy [6].

One discussion regarding the first case concerns the approach to antiviral therapies if the patient had initially presented with all three viruses, with positive viremia, whether the treatments could have been administered concomitantly or in a specific sequence. Another aspect of this case is whether the patient was infected with all three viruses simultaneously or separately, and whether their immune system achieved spontaneous clearance of HBV and HDV, or if the Pegylated interferon α and ribavirin therapy from 2001 facilitated this clearance. There are no available paraclinical evaluation records from that period in order to confirm this. A documented history of HCV viremia spanning a minimum of 23 years could have contributed to the patient’s high fibrosis level, but the combined action of all three hepatitis viruses before the clearance of HBV and HDV might also be a factor. The favorable post-DAA therapy evolution—HCV clearance, biochemical normalization, fibrosis regression by one stage (according to FibroMax), and the reduction of hepatic inflammation and steatosis—significantly improves the patient’s prognosis. However, the patient is treated with silymarin and essential amino acids, follows a hepatoprotective diet, and is monitored clinically every 6 months due to the high amount of fibrosis and the danger of hepatocellular cancer.

The natural progression of HDV towards liver cirrhosis, the spontaneous clearance of HCV in the absence of specific antiviral treatment, and the excellent response to Bulevirtide and Entecavir therapy—with a notable improvement in ALT and AST values after 4 weeks of treatment and no reported side effects—are the main topics of discussion in relation to the second case, which has HDV dominance.

The high severity of cases with triple infection has sparked our interest in the concomitant antiviral treatment for all three viruses.

From the perspective of drug interactions (Table 4), according to the University of Liverpool’s database [35,36], we can administer Bulevirtide concomitantly with Entecavir, Tenofovir alafenamide, Adefovir, and Sofosbuvir–Velpatasvir with no expected interactions. Additionally, Bulevirtide can be administered with Glecaprevir–Pibrentasvir or with Sofosbuvir–Velpatasvir–Voxilaprevir with a potential weak interaction. Additionally, there are no anticipated interactions when Glecaprevir–Pibrentasvir, Sofosbuvir–Velpatasvir, or Sofosbuvir–Velpatasvir–Voxilaprevir are administered concurrently with Entecavir, Tenofovir alafenamide, and Adefovir (Table 5) [35,36].

From the standpoint of medication interactions, it is, therefore, possible to conclude that individuals with triple infection (HBV, HCV, and HDV) have significant liver damage, which supports the necessity of early antiviral treatment utilizing a triple-drug regimen.

The low number of dual and triple infection cases and the lack of treatment experience in treating patients with replicative triple infection, the Romanian cohort from a single centre, and results without multivariable adjustments are the primary limitations of our investigation. Another limitation of our study is the fact that we have excluded all cases with decompensated cirrhosis, from all groups, all etiologies, because these cases benefit from treatment in another clinic, and we do not have data on this pathology segment.

The high severity of triple-infection cases and their citation in the literature, particularly in geographical areas with high endemicity for HCV or HBV, suggests the need for future clinical studies to demonstrate the efficacy and effectiveness of triple antiviral therapy regimens using currently available molecules.

Innovative antiviral therapies targeting HBsAg clearance and the possibility of maintaining undetectable HDV RNA during BLV treatment open new treatment options for patients with autoimmune pathology and co-infected with HDV and HBV, who will be able to safely benefit from powerful immunosuppressive therapies [37,38].

## 5. Conclusions

Concomitant therapy with Bulevirtide, DAA, and NUCs is feasible, and therapeutic outcomes from clinical studies on patients with single infections are promising for improving the prognosis of these patients.

Our small cohort suggests that dual and triple infections present with more advanced liver disease at therapy initiation. Larger multicentre studies are needed to confirm the safety and efficacy of simultaneous BLV + DAA + NUC regimens.

## Figures and Tables

**Table 1:** Fibro Scan interpretation values for each type of viral etiology of chronic hepatitis [32,33].

	F0–F1	F2	F3	F4
HBV [32]	2–7 kPa	7.1–9.4 kPa	9.5–12.4 kPa	≥12.5 kPa
HCV [33]	2–7 kPa	7.1–9.4 kPa	9.5–12.4 kPa	≥12.5 kPa

**Table 2:** (**a**). Comparative analysis of age and ALT, AST, TBR, and DBR in patients with hepatic single infections versus hepatic co-infections. (**b**). Comparative analysis of INR, Albumine, AFP, and Platelet in patients with hepatic single infections versus hepatic co-infections.

**(a)**												
**Descriptives**												
**Groups**			**AGE**		**ALT**		**AST**		**TBR**		**DBR**	
			**Statistic**	**Std. Error**	**Statistic**	**Std. Error**	**Statistic**	**Std. Error**	**Statistic**	**Std. Error**	**Statistic**	**Std. Error**
HCV	Mean		65.19	0.482	82.2244	2.7938	67.0825	2.3187	0.7663	0.0146	0.3036	0.0082
	95% CI for Mean	Lower Bound	64.25		76.7380		62.5291		0.7376		0.2875	
		Upper Bound	66.14		87.7108		71.6359		0.7951		0.3198	
	5% Trimmed Mean		65.55		72.8215		59.0505		0.7323		0.2770	
	Median		66.00		61.6000		49.8000		0.6800		0.2500	
	Variance		157.658		4941.02		3403.41		0.136		0.043	
	SD		12.556		70.2924		58.3388		0.3682		0.2067	
	Minimum		13		10.30		15.00		0.15		0.03	
	Maximum		91		573.30		735.30		3.00		2.71	
	Range		78		563.00		720.30		2.85		2.68	
	Interquartile Range		17		60.20		44.50		0.38		0.15	
	Skewness		−0.473	0.094	2.798	0.097	4.406	0.097	2.139	0.097	4.714	0.097
	Kurtosis		0.178	0.187	10.851	0.194	33.416	0.194	8.003	0.194	37.463	0.194
HBV	Mean		59.25	1.345	45.0080	6.4214	41.7417	4.0179	0.8964	0.0669	0.2969	0.0294
	95% Confidence Interval for Mean	Lower Bound	56.58		32.2131		33.7359		0.7630		0.2383	
		Upper Bound	61.92		57.8029		49.7476		1.0298		0.3556	
	5% Trimmed Mean		59.63		36.9526		37.6693		0.8209		0.2571	
	Median		61.00		27.5000		27.5000		0.7500		0.2200	
	Variance		173.600		3092.57		1210.77		0.336		0.065	
	SD		13.176		55.6109		34.7961		0.5796		0.2548	
	Minimum		24		11.20		1.23		0.24		0.08	
	Maximum		84		428.00		165.10		3.23		1.41	
	Range		60		416.80		163.87		2.99		1.33	
	Interquartile Range		21		34.20		22.30		0.41		0.12	
	Skewness		−0.448	0.246	4.854	0.277	1.939	0.277	2.276	0.277	2.834	0.277
	Kurtosis		−0.497	0.488	30.607	0.548	3.183	0.548	5.809	0.548	8.365	0.548
HCV+ HBV	Mean		67.84	2.807	71.5833	10.6181	57.1583	7.3783	0.8125	0.06987	0.3163	0.0281
	95% CI for Mean	Lower Bound	62.05		49.6181		41.8950		0.6680		0.2580	
		Upper Bound	73.63		93.5486		72.4217		0.9570		0.3745	
	5% Trimmed Mean		68.04		67.5213		54.5528		0.7930		0.3051	
	Median		70.00		50.8500		46.0500		0.7000		0.2700	
	Variance		196.973		2705.87		1306.57		0.117		0.019	
	SD		14.035		52.0180		36.1465		0.34228		0.13802	
	Minimum		41		17.40		17.20		0.31		0.13	
	Maximum		91		205.10		146.70		1.69		0.72	
	Range		50		187.70		129.50		1.38		0.59	
	Interquartile Range		25		63.28		55.73		0.38		0.18	
	Skewness		−0.400	0.464	1.138	0.472	1.099	0.472	1.044	0.472	1.326	0.472
	Kurtosis		−0.823	0.902	0.579	0.918	0.292	0.918	0.952	0.918	2.245	0.918
HVD + HBV	Mean		57.29	3.050	96.2786	10.4725	89.3286	13.5468	1.0579	0.1350	0.3643	0.0701
	95% CI for Mean	Lower Bound	50.70		73.6540		60.0624		0.7661		0.2128	
		Upper Bound	63.87		118.9031		118.5948		1.3496		0.5158	
	5% Trimmed Mean		57.60		95.3040		84.2540		1.0321		0.3364	
	Median		61.00		92.5500		74.5000		0.8750		0.2550	
	Variance		130.220		1535.43		2569.23		0.255		0.069	
	SD		11.411		39.1846		50.6876		0.50534		0.26235	
	Minimum		36		46.30		37.20		0.51		0.15	
	Maximum		73		163.80		232.80		2.07		1.08	
	Range		37		117.50		195.60		1.56		0.93	
	Interquartile Range		22		81.48		44.15		0.57		0.23	
	Skewness		−0.585	0.597	0.344	0.597	1.958	0.597	1.274	0.597	1.969	0.597
	Kurtosis		−0.891	1.154	−1.209	1.154	4.507	1.154	0.255	1.154	3.576	1.154
**(b)**												
**Descriptives**												
					**INR**		**ALB**		**AFP**		**Platelet**	
					**Statistic**	**Std. Error**	**Statistic**	**Std. Error**	**Statistic**	**Std. Error**	**Statistic**	**Std. Error**
HCV	Mean				1.0725	0.0143	4.8651	0.3571	4.6970	1.2741	205.2929	4.5549
	95% CI for Mean		Lower Bound		1.0443		4.1622		2.1894		196.3287	
			Upper Bound		1.1007		5.5679		7.2046		214.2571	
	5% Trimmed Mean				1.0375		4.5248		2.8348		202.3475	
	Median				1.0200		4.5100		2.5300		200.0000	
	Variance				0.061		37.879		482.186		6162.019	
	SD				0.2468		6.1546		21.9587		78.4985	
	Minimum				0.77		2.91		0.50		33.00	
	Maximum				2.72		110.40		360.63		531.00	
	Range				1.95		107.49		360.13		498.00	
	Interquartile Range				0.14		0.43		2.18		95.50	
	Skewness				4.471	0.141	17.148	0.141	15.001	0.141	0.746	0.141
	Kurtosis				23.809	0.282	295.021	0.282	237.741	0.282	1.759	0.282
HBV	Mean				1.0941	0.03544	4.3880	0.06680	5.9434	3.11495	211.8409	15.82749
	95% CI for Mean		Lower Bound		1.0226		4.2532		−0.3385		179.9217	
			Upper Bound		1.1656		4.5227		12.2253		243.7601	
	5% Trimmed Mean				1.0575		4.4157		2.6360		204.7778	
	Median				1.0400		4.4350		2.1600		211.0000	
	Variance				0.055		0.196		426.929		11,022.416	
	SD				0.2350		0.4430		20.6622		104.9876	
	Minimum				0.79		2.93		0.51		3.00	
	Maximum				2.06		5.08		138.79		598.00	
	Range				1.27		2.15		138.28		595.00	
	Interquartile Range				0.14		0.50		1.60		82.00	
	Skewness				3.154	0.357	−1.092	0.357	6.471	0.357	1.230	0.357
	Kurtosis				11.113	0.702	1.648	0.702	42.475	0.702	3.974	0.702
HCV + HBV	Mean				1.0606	0.02222	4.4265	0.06354	3.4929	0.65489	201.5882	14.82894
	95% CI for Mean		Lower Bound		1.0135		4.2918		2.1046		170.1523	
			Upper Bound		1.1077		4.5612		4.8813		233.0242	
	5% Trimmed Mean				1.0618		4.4427		3.1738		201.2647	
	Median				1.0800		4.4800		2.4600		196.0000	
	Variance				0.008		0.069		7.291		3738.257	
	SD				0.0916		0.2619		2.7001		61.1412	
	Minimum				0.90		3.81		0.60		84.00	
	Maximum				1.20		4.75		12.13		325.00	
	Range				0.30		0.94		11.53		241.00	
	Interquartile Range				0.17		0.27		2.61		79.50	
	Skewness				−0.238	0.550	−1.081	0.550	2.148	0.550	0.346	0.550
	Kurtosis				−1.195	1.063	0.722	1.063	6.224	1.063	0.018	1.063
HVD + HBV	Mean				1.1300	0.04240	4.2746	0.15194	7.3631	2.35005	127.4615	17.60886
	95% CI for Mean		Lower Bound		1.0376		3.9436		2.2428		89.0951	
			Upper Bound		1.2224		4.6057		12.4834		165.8280	
	5% Trimmed Mean				1.1311		4.2662		6.6923		125.0684	
	Median				1.1200		4.2500		3.2500		125.0000	
	Variance				0.023		0.300		71.796		4030.936	
	SD				0.1528		0.5478		8.4732		63.4896	
	Minimum				0.88		3.33		2.09		46.00	
	Maximum				1.36		5.37		24.71		252.00	
	Range				0.48		2.04		22.62		206.00	
	Interquartile Range				0.28		0.81		8.24		102.00	
	Skewness				−0.021	0.616	0.226	0.616	1.548	0.616	0.494	0.616
	Kurtosis				−1.282	1.191	0.180	1.191	0.673	1.191	−0.633	1.191

Legend: SD = Standard Deviation; CI = Confidence Interval. The normal values are: ALT—0–45 U/L, AST—0/35 U/L, Total Bilirubin (TBR)—0.1–1.2 mg/dL, Direct bilirubin (DBR)—0–0.2 mg/dL. Platelets—150–450 × 10^3^/μL, INR—0.80–1.20, serum albumin (ALB)—3.5–5.5 g/dL, AFP—1–10 UI/mL.

**Table 3:** Characteristics of categorical variables in patients with hepatic single infections versus hepatic co-infections.

	HCV (N1 = 679)		HBV (N2 =98)		HCV + HBV (N3 = 25)		HDV + HBV (N4 = 14)	
	*n*	%	*n*	%	*n*	%	*n*	%
Sex								
Female	464	68.33	33	33.67	20	80	6	42.85
Male	195	31.66	65	66.32	5	20	8	57.14
Living Area								
Urban	372	54.78	72	73.46	14	56	9	64.28
Rural	307	45.21	26	26.53	11	44	5	35.71
Liver fibrosis								
F0-F1	133	19.58	39	39.79	4	16	0	0
F2	251	36.96	27	27.55	3	12	3	21.42
F3	134	19.73	13	13.26	15	60	4	28.57
F4	161	23.71	19	19.38	3	12	7	50

Legend: Liver fibrosis—F-(kPa-Fibroscan) [32,33].

**Table 4:** Bulevirtide drug interaction with: Adefovir, Entecavir, Glecaprevir/Pibrentasvir, Sofosbuvir/Velpatasvir, Sofosbuvir/Velpatasvir/Voxilaprevir, and Tenofovir alafenamide [35,36].

	Bulevirtide
Adefovir	No Interaction Expected
Entecavir	No Interaction Expected
Tenofovir alafenamide	No Interaction Expected
Glecaprevir/Pibrentasvir	Potential Weak Interaction
Sofosbuvir/Velpatasvir	No Interaction Expected
Sofosbuvir/Velpatasvir/Voxilaprevir	Potential Weak Interaction

**Table 5:** Glecaprevir/Pibrentasvir, Sofosbuvir/Velpatasvir, and Sofosbuvir/Velpatasvir/Voxilaprevir drug interaction with Adefovir, Entecavir, and Tenofovir alafenamide [35,36].

	Glecaprevir/Pibrentasvir	Sofosbuvir/Velpatasvir	Sofosbuvir/Velpatasvir/Voxilaprevir
Adefovir	No Interaction Expected	No Interaction Expected	No Interaction Expected
Entecavir	No Interaction Expected	No Interaction Expected	No Interaction Expected
Tenofovir alafenamide	No Interaction Expected	No Interaction Expected	No Interaction Expected

## Data Availability

Data Availability Statements are available on request through the corresponding author.
